# The effectiveness of mindful walking based on the timing it right framework in patients with atrial fibrillation and chronic heart failure

**DOI:** 10.3389/fcvm.2025.1587547

**Published:** 2025-06-19

**Authors:** Qin Lu, Jingjing Lu, Yeping Zheng, Juanqin Shen, Xia Zhao, Jianjiang Xu, Xiaoping Gu, Zhenliang Chu

**Affiliations:** ^1^ Department of Cardiology, The Second Affiliated Hospital of Jiaxing University, Jiaxing, Zhejiang, China; ^2^ Department of Nursing, Sunto Women and Children’s Hospital, Jiaxing, Zhejiang, China

**Keywords:** atrial fibrillation, chronic heart failure, mindful walking, negative emotions, timing it right framework

## Abstract

**Objective:**

To explore the effects of mindful walking (MFW) based on the Timing It Right (TIR) framework on anxiety, depression, and cardiopulmonary function in patients with atrial fibrillation combined with chronic heart failure.

**Methods:**

A total of 86 patients with atrial fibrillation and chronic heart failure admitted to the cardiology department of our hospital between June 2023 and March 2024 were enrolled in this study. Two homogeneous management wards were selected, with 43 patients from Ward A serving as the control group and 43 patients from Ward B as the intervention group. After excluding those lost to follow-up or readmitted, 39 patients were included in the control group and 41 in the intervention group. The control group received routine care, discharge guidance, and exercise rehabilitation training, while the intervention group received MFW intervention based on the TIR framework in addition to the standard care treatment. The Five Facet Mindfulness Questionnaire (FFMQ), Self-Rating Anxiety Scale (SAS), Self-Rating Depression Scale (SDS), 6-minute Walk Distance (6MWD), incidence of adverse cardiac events, and Senior Fitness Test (SFT) were used for effect evaluation.

**Results:**

The observation period of the study was 24 weeks, and there were no significant differences in general information, pre-intervention SAS and SDS scores, FFMQ, 6MWD, and SFT between the two groups (all *P* > 0.05). Post-intervention, the SAS and SDS scores in the intervention group were 38.12 ± 4.18 and 37.34 ± 3.62, significantly lower than the control group scores of 54.05 ± 5.93 and 51.15 ± 5.91 (*t* = 13.938, 12.679, both *P* < 0.001). The intervention group's FFMQ scores for observing, describing, and acting with awareness were 25.98 ± 4.14, 28.68 ± 3.12, and 32.02 ± 3.49, all significantly higher than the control group's scores of 20.85 ± 3.31, 22.41 ± 1.94, and 26.82 ± 3.75 (*t* = −6.101, −10.735, −6.431, all *P* < 0.001). The 6MWD for the intervention group was greater than the control group (376.90 ± 42.99 vs. 312.13 ± 15.01, *t* = −8.904, *P* < 0.001). Additionally, the intervention group's lower limb muscle strength and grip strength were 11.00 ± 2.19 and 22.90 ± 3.94, both superior to the control group's 6.38 ± 2.54 and 16.85 ± 4.59, with significant differences (*t* = −8.715, −7.746, *P* < 0.001).

**Conclusion:**

In patients with atrial fibrillation and chronic heart failure, the Timing It Right-based mindful walking intervention group demonstrated significant improvements in mindfulness capacity, cardiopulmonary performance metrics, and negative emotion scores compared to standard care controls.

## Introduction

Atrial fibrillation (AF) is one of the most prevalent arrhythmias globally, currently affecting an estimated 33.5 million individuals. Projections indicate that by 2060, the number of people living with AF could double (1). Clinically, chronic heart failure (CHF) and AF frequently coexist. Persistent AF can exacerbate symptoms of heart failure (HF) and further deteriorate cardiac function. Conversely, recurring episodes of HF can induce atrial enlargement, fostering the progression of AF, creating a vicious cycle between the two conditions (2). At present, exercise-based rehabilitation is recommended as a Class I, Level A treatment for patients with both AF and CHF by cardiac rehabilitation specialists in both Western countries and China (3–5). Mindfulness-based interventions (6), a safe and non-pharmacological therapeutic approach that incorporates physical activity, have been shown to significantly alleviate negative emotions (7). One such intervention, mindful walking (MFW), combines physical exercise with meditation (8), and has been demonstrated to enhance patients' emotional well-being and improve cardiopulmonary function.

The Timing It Right (TIR) framework, introduced by Cameron et al. (9) in 2008 in the context of caregiving for stroke patients, segments the disease or treatment process into several stages: diagnostic, stable, preparatory, action, and adaptation. Each stage addresses the evolving needs for information, emotional support, tools, and evaluation, ensuring that the changing demands of both patients and caregivers are met. This rehabilitation model, based on the TIR framework, has been applied primarily to coronary heart disease and chronic obstructive pulmonary disease, yielding positive outcomes in patient recovery (10). In recent years, the TIR framework has been utilized across various disease studies, yielding promising results by tailoring interventions according to the specific characteristics of each disease stage. Additionally, the TIR framework promotes a smooth transition from hospital to home, offering a solid theoretical foundation for cardiac rehabilitation. The present study aims to investigate the impact of MFW, based on the TIR framework, in patients with both AF and CHF, as detailed below.

## Methods

### Study design

The current study compared the effectiveness of MFW based on TIR framework in patients with AF and CHF who were admitted to the cardiology department of our hospital between June 2023 and March 2024. Participants were randomly assigned to either the intervention or control groups via a computer-generated random number table with allocation concealment maintained by sequentially numbered opaque envelopes. AF was defined as patients with a standard electrocardiogram (ECG) or 24-h Holter monitoring indicating persistent atrial fibrillation (11). The study was blinded only to outcome evaluators, data monitors, and statistical analysts. CONSORT guidelines for reporting randomized trials were applied. The study was approved by the hospital's medical ethics committee (2023JX006-01). The observation period of the study was 24 weeks.

### Participants

Sample Size Estimation Formula:

$$N={\left[\frac{Z\alpha /2\sqrt{\pi c(1-\pi c)(Q1^{-1}+Q2^{-1})}+Z\beta \sqrt{\pi 1(1-\pi 1)/Q1+\pi 2(1-\pi 2)/Q2}}{\pi 1-\pi 2}\right]}^{2}$$

Based on the sample size calculation formula for comparing two independent sample rates (12), with a 95% confidence interval and a 5% margin of error, the baseline incidence of adverse events in cardiac rehabilitation was estimated at 27% (13), and the anticipated reduction in the incidence of adverse events was 7%. The calculated sample size for this study is 86 cases. To avoid contamination of the sample, two homogenized wards were selected.

Inclusion Criteria:

1. Age ≥18 years.
2. Meeting the 2018 Chinese diagnostic criteria for heart failure (14).
3. New York Heart Association (NYHA) functional class II–III, Heart failure with preserved ejection fraction (HFpEF).
4. Presence of negative emotions with Self-Rating Anxiety Scale (SAS) score ≥50 or Self-Rating Depression Scale (SDS) score ≥50).
5. No history of regular exercise (defined as exercising ≥3 times per week for ≥30 min each time).
6. No severe dysfunction of major organs (brain, liver, lungs, kidneys).
7. Fully functional lower limbs and no regular exercise history.
8. Full cognitive and behavioral capacity.
9. Signed informed consent.

Exclusion Criteria:

1. Patients with contraindications to cardiac rehabilitation exercise testing and training.
2. Patients who voluntarily withdrew from the study for any reason.
3. Patients unable to comprehend the questionnaire.
4. Patients readmission within 24 weeks.

### Intervention

#### Control group

Patients in the control group received routine discharge care, discharge guidance, cardiac rehabilitation education, and exercise training guidance within 3 days before discharge. The content included:

1. Formation of an Intervention Team: The team consisted of one cardiac rehabilitation physician, three cardiac rehabilitation nurses, one cardiac rehabilitation therapist, and one psychological counselor. All team members had over five years of experience in cardiac rehabilitation guidance and underwent standardized training for the project with clearly defined responsibilities.
2. Explanation of the Importance of Cardiac Rehabilitation: Patients and their families were educated on the importance of cardiac rehabilitation training and encouraged to actively participate in rehabilitation exercises. Patients were advised to choose exercise types based on their condition, such as walking, Tai Chi, or Baduanjin, with a focus on exercise precautions. However, no specific requirements were made regarding the intensity or duration of the exercise.
3. Specialist Outpatient Follow-up: Patients had one specialist outpatient follow-up two weeks after discharge and monthly follow-ups via telephone thereafter.

#### Intervention group

In addition to the measures provided to the control group, the intervention group received MFW interventions based on the TIR framework (15), shown as Supplementary Figure S1. The specific contents were as follows:

1. Exercise on a Quiet Road.
2. 2. Walking with Pauses: After every 10–15 steps, patients were instructed to pause. The duration of the pause could be adjusted based on personal preference before resuming walking.
3. Mindful Movement: While walking, attention was focused on every movement of the body: lifting the foot, the movement of the foot in the air, the landing of the foot, and the shifting of the body's center of gravity.
4. Natural Walking Pace: Maintain a natural walking pace without deliberately trying to walk faster or slower.
5. Hand Position: Hands can be placed behind the back or allowed to swing naturally.
6. Breathing and Balance: Focus on your breathing and the balance of your head, keeping your eyes looking forward.
7. Refocusing: If attention or thoughts drift, gently bring them back to the current movements and sensations.
8. Integration into Daily Life: Once mindful walking becomes more familiar, try to integrate it into daily life.

Additionally, patients were educated on emergency handling methods in case of discomfort during exercise and encouraged to seek medical attention if necessary. Patients were instructed to log their daily exercise data, such as heart rate, blood pressure, and exercise intensity, via WeChat check-ins or app screenshots. If two consecutive reports were missed in a week, an explanation was required. Follow-up via phone or WeChat was conducted to assess exercise tolerance and adherence, addressing any concerns.

### Outcome measures

The outcome indicators of the two groups were measured before the study began and 24 weeks after the intervention (Supplementary Figure S1).

#### Negative emotions

Negative emotions were measured before and after the intervention using the Self-Rating Anxiety Scale (SAS) and the Self-Rating Depression Scale (SDS). The SAS, developed in 1971 (16), has a reliability of 0.824 and validity of 0.934. It consists of 20 items rated on a 4-point Likert scale: “none or rarely,” “sometimes,” “most of the time,” and “almost or all the time,” scored from 1 to 4, respectively. Fifteen items are negative statements, scored positively, while the remaining five are positive statements, scored inversely. The total score ranges from 20 to 80, with a score of ≥50 indicating anxiety; the higher the score, the more severe the anxiety (17). The SDS, also developed by Zung in 1965 (16), has a reliability of 0.819 and a validity of 0.916. It consists of 20 items rated on a 4-point Likert scale. Ten items are negative statements, scored positively, and ten are positive statements, scored inversely. The total score ranges from 20 to 80, with a score of ≥53 indicating depression; the higher the score, the more severe the depression (18).

#### Five facet mindfulness questionnaire (FFMQ)

The FFMQ was used to assess the overall level of mindfulness in patients before and after the intervention. It was developed by Baer et al. (19), and the Chinese version used in this study was revised by Deng et al. (20). The questionnaire consists of 39 items and measures five dimensions: observing, describing, acting with awareness, non-judging, and non-reactivity. It is scored on a 5-point Likert scale, with a total score ranging from 39 to 195. Scores for observing, describing, acting with awareness, and non-judging range from 8 to 40, while non-reactivity ranges from 7 to 35. Higher scores indicate higher levels of mindfulness. The Cronbach's alpha coefficient for the FFMQ's dimensions ranges from 0.792 to 0.905 (21).

#### Cardiopulmonary function assessment

Cardiopulmonary function was assessed using the 6-minute walk distance (6MWD) before and after the intervention (22).

#### Adverse cardiac events

Adverse cardiac events included serious complications such as malignant arrhythmias, angina, HF and cardiac arrest that occurred after the intervention.

#### Functional fitness

Functional fitness was measured using the Senior Fitness Test (SFT) developed by Rikli et al. (23). This test is safe, simple, and easy to perform, and includes the following:

Lower limb muscle strength: Participants sat upright in a chair with arms crossed over their chest. The test began in a seated position, and the participant stood up and sat down as many times as possible within 30 s.

Upper limb flexibility: Measured using the back scratch test, where participants reached behind their back to touch or overlap their hands, holding the position for at least 2 s. The distance between the middle fingertips was measured, with positive values indicating overlap and negative values indicating a gap.

Lower limb flexibility: Measured using the chair sit-and-reach test, where participants sat on a 43 cm-high chair, extended their dominant leg, and reached forward as far as possible. The distance between the middle fingertip and toe was recorded, with positive values indicating the fingertip exceeded the toe, and negative values indicating it fell short.

Handgrip strength: Measured using a Chinese Xiangshan EH101 electronic handgrip dynamometer. Participants stood upright with their feet shoulder-width apart and arms hanging naturally while gripping the device as hard as possible for 5 s. The test was repeated twice, with a 30-s interval, and the maximum value was recorded.

#### Cardiac rehabilitation satisfaction

Cardiac rehabilitation satisfaction was assessed using a self-made patient satisfaction survey after the intervention, which evaluates five dimensions: service attitude, health education, team professionalism, cardiac rehabilitation equipment and tools, and cardiac rehabilitation treatment arrangements. Each item is scored from 1 to 10, with a total score of 100 points. A higher score indicates greater patient satisfaction with the rehabilitation. The Cronbach's *α* coefficient was 0.85. Scores above 95 points indicate “very satisfied,” scores between 80 and 95 indicate “satisfied,” and scores below 80 indicate “dissatisfied.” Nursing satisfaction is calculated as the ratio of very satisfied and satisfied cases to the total number of cases, multiplied by 100%.

### Data collection and management

Before the study, the research team underwent standardized training to clarify responsibilities and understand key considerations, ensuring the accuracy of intervention methods and survey results. During the study, researchers distributed questionnaires one-on-one in person, using standardized language to avoid leading questions. Measurements were taken strictly according to the guidelines to ensure data accuracy. The questionnaires were collected and checked on-site, and incomplete questionnaires were addressed immediately to ensure full data collection. Questionnaires missing essential information were excluded.

### Statistical methods

Data analysis was performed using SPSS 26.0 statistical software. Quantitative data were expressed as mean ± standard deviation, with intergroup comparisons made using the *t*-test, and intragroup comparisons made using the paired *t*-test. Categorical data were expressed as frequencies and percentages (%), and intergroup comparisons were made using the *χ*² test. All tests were two-sided, with *P* < 0.05 indicating statistical significance.

## Results

**Figure 1:**
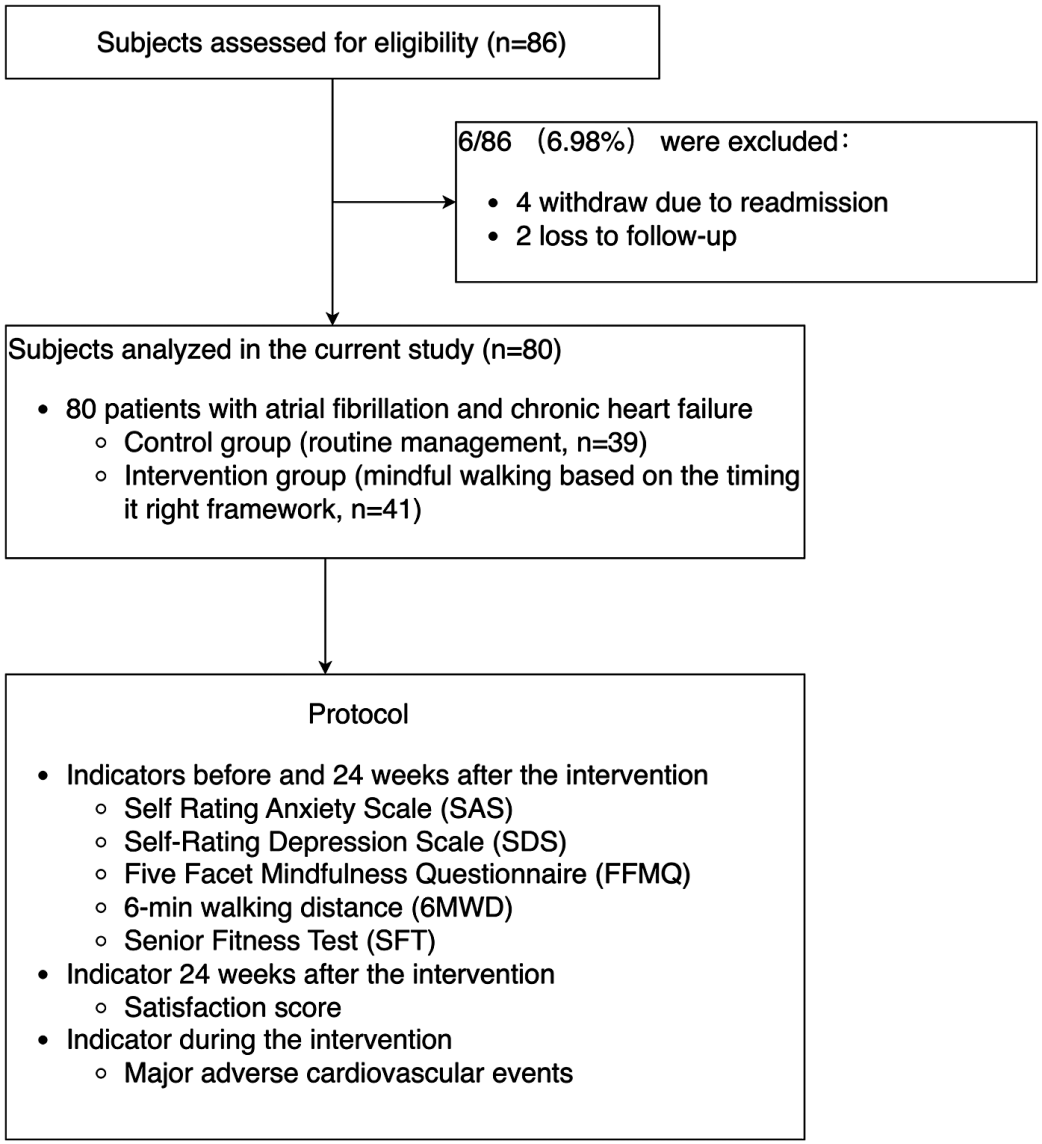
Enrolment flow diagram and the protocol.

A total of 86 eligible patients were identified. Of these, 43 patients from Cardiovascular Ward A were assigned to the control group, and 43 patients from Cardiovascular Ward B were assigned to the intervention group. During follow-up, 4 patients were lost from the control group (due to rehospitalization and voluntary withdrawal), and 2 patients were lost from the intervention group. Therefore, a total of 80 patients completed the study: 39 in the control group and 41 in the intervention group (Figure 1). There were no statistically significant differences in baseline characteristics between the two groups (*P* > 0.05), making them comparable. The Baseline characteristics were shown in Table 1.

**Table 1:** Baseline characteristics between the two groups.

Baseline characteristics		Control (*n* = 39)	Intervention (*n* = 41)	*t*/*χ*^2^	*p*
Gender [*n* (%)]	Male	23 (58.97)	19 (46.34)	1.279	0.258
	Female	16 (41.03)	22 (53.66)		
Age (years)		56.90 ± 10.23	57.00 ± 10.45	−0.044	0.965
Education level [*n *(%)]	Primary school and below	20 (51.28)	16 (39.02)	1.213	0.271
	Junior high school and above	19 (48.72)	25 (60.98)		
Duration of diseases (years)		16.08 ± 7.63	16.95 ± 8.01	−0.499	0.619
Types of chronic diseases [*n *(%)]	Diabetes	6 (15.38)	7 (17.07)	0.042	0.838
	Hypertension	16 (41.03)	15 (36.58)	0.166	0.684
	Chronic renal insufficiency	6 (15.38)	6 (14.63)	0.009	0.925
New York Heart Association functional class [*n *(%)]	II	16 (41.03)	19 (46.34)	0.230	0.632
	III	23 (58.97)	22 (53.66)		
Guideline-directed medical therapy (GDMT)	YES	25 (64.10)	23 (56.10)	0.534	0.465
	NO	14 (35.90)	18 (43.90)		
Medication compliance	YES	23 (58.97)	31 (75.61)	2.521	0.112
	NO	16 (41.03)	10 (24.39)		

### Comparison of negative emotion scores, 6MWD, and functional fitness between the two groups

After intervention, the SAS and SDS scores of intervention group decreased, with the intervention group showing significantly lower scores than the control group (*P* < 0.001).The 6MWD in the intervention group was significantly greater than in the control group after intervention (*P* < 0.001).The improvements in handgrip strength and lower limb muscle strength in the intervention group were significantly greater than those in the control group after intervention (*P* < 0.001),as shown in Table 2.

**Table 2:** Comparison of negative emotion scores, 6MWD, functional fitness between the Two groups $\left(\bar{x}\pm s\right)$.

Observation indicator	Before intervention	After intervention	*t/χ* ^2^	*p*
SAS (points)				
Control (*n* = 39)	56.41 ± 6.04	54.05 ± 5.93	1.703	0.097
Intervention (*n* = 41)	56.12 ± 5.90	38.12 ± 4.18	15.101	<0.001
*T*	0.216	13.938		
*P*	0.830	<0.001		
SDS (points)				
Control (*n* = 39)	53.05 ± 5.15	51.15 ± 5.91	1.464	0.151
Intervention (*n* = 41)	54.07 ± 6.33	37.34 ± 3.62	15.415	<0.001
*T*	−0.790	12.679		
*P*	0.432	<0.001		
6MWD (m)				
Control (*n* = 39)	302.33 ± 15.78	312.13 ± 15.01	−2.695	<0.05
Intervention (*n* = 41)	303.66 ± 15.17	376.90 ± 42.99	−9.404	<0.001
*T*	−0.383	−8.904		
*P*	0.703	<0.001		
Lower limb muscle strength (times/30 s)				
Control (*n* = 39)	5.51 ± 1.97	6.38 ± 2.54	−2.041	<0.05
Intervention (*n* = 41)	6.11 ± 1.86	11.00 ± 2.19	−11.371	<0.001
*t*	−1.537	−8.715		
*p*	0.128	<0.001		
Upper limb flexibility (cm)				
Control (*n* = 39)	−17.10 ± 3.31	−17.79 ± 3.34	−0.881	0.384
Intervention (*n* = 41)	−18.66 ± 3.66	−18.66 ± 3.27	0.000	1.000
*t*	−1.992	−1.169		
*p*	0.050	0.246		
Lower limb flexibility (cm)				
Control (*n* = 39)	−15.90 ± 4.78	−15.38 ± 6.44	0.429	0.670
Intervention (*n* = 41)	−14.88 ± 5.29	−14.80 ± 5.72	0.067	0.947
*t*	0.903	0.426		
*p*	0.369	0.671		
Handgrip strength (kg)				
Control (*n* = 39)	16.36 ± 5.06	16.85 ± 4.59	−0.423	0.675
Intervention (*n* = 41)	17.24 ± 3.46	22.90 ± 3.94	−7.746	<0.001
*t*	−0.917	−7.746		
*p*	0.362	<0.001		

SAS, Self-Rating Anxiety Scale; SDS, Self-Rating Depression Scale; 6MWD, 6-minute walk distance.

### Comparison of five facet mindfulness questionnaire (FFMQ) scores between the two groups

Before the intervention, there were no statistically significant differences between the two groups in any of the five dimensions (*P* > 0.05). After the intervention, there were significant differences in the observing, describing, and acting with awareness dimensions, with the intervention group scoring significantly higher than the control group (all *P* < 0.001). Although the intervention group scored higher than the control group in the non-reactivity and non-judging dimensions, the differences were not statistically significant (*P* > 0.05), as shown in Table 3.

**Table 3:** Comparison of five facet mindfulness questionnaire scores between the Two groups (points, $\bar{x}\pm s$).

FFMQ scores	Before intervention	After intervention	*t*	*p*
FFMQ				
Control (*n* = 39)	107.36 ± 10.78	106.74 ± 7.56	0.278	0.783
Intervention (*n* = 41)	110.22 ± 10.35	123.66 ± 12.37	−5.228	<0.001
*t*	−1.211	−7.337		
*p*	0.230	<0.001		
Observing				
Control (*n* = 39)	20.23 ± 2.58	20.85 ± 3.31	−1.005	0.321
Intervention (*n* = 41)	19.78 ± 1.86	25.98 ± 4.14	−9.027	<0.001
*t*	0.898	−6.101		
*p*	0.372	<0.001		
Describing				
Control (*n* = 39)	22.38 ± 1.98	22.41 ± 1.94	−0.060	0.952
Intervention (*n* = 41)	21.76 ± 2.18	28.68 ± 3.12	−11.151	<0.001
*t*	1.348	−10.735		
*p*	0.181	<0.001		
Acting with awareness				
Control (*n* = 39)	27.69 ± 4.08	26.82 ± 3.75	1.057	0.297
Intervention (*n* = 41)	27.51 ± 3.64	32.02 ± 3.49	−6.037	<0.001
*t*	0.209	−6.431		
*p*	0.835	<0.001		
Non-judging				
Control (*n* = 39)	23.41 ± 2.07	24.26 ± 2.05	−1.796	0.081
Intervention (*n* = 41)	23.88 ± 2.45	23.95 ± 1.84	−0.148	0.883
*t*	−0.919	0.701		
*p*	0.361	0.485		
Non-reactivity				
Control (*n* = 39)	19.97 ± 3.73	21.15 ± 2.39	−1.781	0.083
Intervention (*n* = 41)	20.98 ± 3.22	21.29 ± 2.72	−0.467	0.643
*t*	−1.287	−0.242		
*p*	0.202	0.809		

FFMQ, Five Facet Mindfulness Questionnaire.

### Comparison of adverse cardiac events between the two groups

There was no significant difference in the incidence of adverse cardiac events between the two groups (*P* > 0.05), as shown in Table 4.

**Table 4:** Comparison of adverse cardiac events between the Two groups [*n* (%)].

Adverse cardiac events	Before intervention	After intervention	*t/χ* ^2^	*p*
Malignant arrhythmia				
Control (*n* = 39)	1 (2.56)	0 (0)		
Intervention (*n* = 41)	0 (0)	0 (0)		
Angina				
Control (*n* = 39)	3 (7.69)	1 (2.56)		
Intervention (*n* = 41)	1 (2.44)	1 (2.44)		
Heart failure				
Control (*n* = 39)	4 (10.26)	2 (5.13)		
Intervention (*n* = 41)	4 (9.76)	1 (2.44)		
Cardiac arrest				
Control (*n* = 39)	0 (0)	0 (0)		
Intervention (*n* = 41)	0 (0)	0 (0)		
Adverse Cardiac Events				
Control (*n* = 39)	8 (20.51)	3 (7.69)	2.646	0.104
Intervention (*n* = 41)	5 (12.20)	2 (4.88)	1.406	0.236
*χ* ^2^	1.016	0.270		
*p*	0.313	0.603		

### Comparison of cardiac rehabilitation satisfaction between the two groups

The cardiac rehabilitation satisfaction rate in the intervention group was 92.68% (38/41), which was higher than the 76.92% (30/39) in the control group, and the difference was statistically significant (*χ*^2^ = 3.894, *P* < 0.05).

### Subgroup analysis results

Subgroup analysis showed no differences in SAS, SDS, 6MWD, and FFMQ across different New York Heart Association functional classes, age groups, and gender (as shown in Supplementary Table S1).

## Discussion

Atrial fibrillation combined with chronic heart failure is characterized by a decline in cardiac function due to cardiovascular diseases, and it is a clinical syndrome resulting from both structural and functional heart diseases. Such patients typically require hospitalization when they are acutely ill and most need to recover at home after their conditions stabilize. After discharge, however, many patients, due to a lack of proper care, experience disruptions in their cardiac rehabilitation process, which adversely affects their prognosis. Therefore, based on the integration of timing theory and mindful walking training, this study found that it helps such patients maintain mindfulness, improve negative emotions and cardiopulmonary function, and does not increase the occurrence of adverse cardiac events, offering an innovative management pathway for patients with complex comorbidities.

### Mindfulness walking based on timing theory helps improve negative emotions in patients

With the advancement of cardiac rehabilitation, psychological factors have become a a critical component. Traditional psychological interventions often focus on verbal comfort, which frequently fails to address deeper psychological concerns. The negative emotions reported by participants in this study primarily stem from the disease itself, including recurrent symptoms such as shortness of breath, fatigue, and reduced exercise tolerance. Furthermore, inadequate knowledge about the disease and cardiac rehabilitation exacerbates uncertainty during treatment, intensifying negative emotions. Mindfulness-based stress reduction (MBSR), originating from mindfulness meditation, has been widely used as an adjunctive therapy for conditions like depression and has been proven effective in alleviating depressive symptoms (24). Rechenberg et al. demonstrated that mindfulness can improve negative emotions and quality of life in CHF patients by helping them overcome automatic negative thinking patterns (25). In this study, after the intervention group underwent mindfulness walking training based on timing theory, their anxiety and depression scores were significantly lower compared to the control group (*P* < 0.001). This suggests that mindfulness walking combined with timing theory can enhances the psychological well-being of patients with AF and CHF. The timing theory divides the rehabilitation process into distinct stages, addressing the evolving needs at each stage, which effectively reduces anxiety and depression (26). Walking, a simple and safe form of rehabilitation exercise, has been shown to improve exercise tolerance in HF patients (27).

### MFW based on TIR framework can improve rehabilitation satisfaction in patients with AF complicated by CHF

Hospital satisfaction is an important indicator of service quality in medical institutions, and team professionalism is a key factor determining hospital satisfaction (28). The results of this study show that the satisfaction scores of the intervention group were significantly higher than those of the control group. This can be attributed to the comprehensive and scientifically designed rehabilitation plan, along with its precise execution by the professional team, which ensured the patients' recovery. The plan closely focused on the patients' psychological state. For patients with negative emotions, psychological intervention was essential for emotional management. At the same time, as disease awareness increased, patients were able to fully understand the development of their condition and simple self-care methods, which helped them view their condition more positively and regulate their emotions. The study showed that after the intervention, the psychological status of the intervention group improved, which is a potential factor for the higher patient satisfaction, leading to a significant increase in satisfaction.

### Mindfulness walking based on timing theory enhances mindfulness awareness

Mindfulness walking, a type of mindfulness-based intervention, integrates gentle physical activity with meditation. As an aerobic exercise, it can be adapted to various environments and paces, allowing individuals to regulate their breathing and focus on their bodily movements without judgment. This fosters a “present moment” experience (29). The findings of this study revealed that mindfulness awareness, particularly in the areas of observing, describing, and acting with awareness, significantly improved in the intervention group (all *P* < 0.001). The overall FFMQ score of the intervention group was also significantly higher than that of the control group (*P* < 0.001). This indicates that the patients in the intervention group were better able to experience and accept their current psychological state. These results align with Ashraf's study, which found that patients with irritable bowel syndrome (IBS) who received mindfulness therapy in addition to medication experienced greater improvements in mindfulness and symptom reduction compared to those who only received medication (30). Mindfulness, a practice rooted in Buddhism, emphasizes non-judgmental awareness of both internal and external stimuli. Mindful walking during meditation helps redirect attention to task-related thoughts, decrease sensory activation, and enhance emotional responses (31). As a cost-effective and non-invasive intervention, mindfulness walking, when combined with timing theory, encourages patients to adopt an accepting, non-resisting attitude toward the pain associated with their condition, improving their ability to live in the present and enhancing their overall outlook (32).

### Mindfulness walking based on timing theory improves cardiopulmonary function and functional fitness

The results indicated that the 6-minute walk distance (6MWD) of patients who participated in mindfulness walking interventions based on timing theory was significantly greater than that of the control group, suggesting improvements in aerobic endurance, consistent with Princ's findings (33). Low levels of functional fitness pose a risk to patients’ ability to perform daily tasks. In this study, no significant differences were observed between the two groups in terms of upper and lower limb flexibility after the intervention (*P* > 0.05). The core goal of mindful walking is to enhance mental concentration, emotional regulation and physical awareness, rather than directly targeting joint range of motion or muscle extensibility. However, significant improvements in handgrip strength and lower limb muscle strength were observed in the intervention group compared to the control group (*P* < 0.001). The tailored guidance provided at different stages of rehabilitation based on timing theory may enhance patient adherence to medical recommendations, encourage healthier lifestyles, mitigate the impact of risk factors on their condition, and help reverse skeletal muscle and vascular abnormalities over time. Long-term cardiac rehabilitation exercises can improve blood flow to muscles, reduce sympathetic nervous system activity (which decreases cardiac afterload), and enhance cardiac function (34).

### Mindfulness walking based on timing theory is feasible

Mindful walking serves as a cost-effective behavioral intervention that requires no specialized equipment or dedicated facilities, making it flexible for implementation in community, residential, or natural environments. This dual-modality approach synergistically combines graded physical activation with psychological regulation, making it particularly suitable for cardiac rehabilitation populations. Clinical observations demonstrate its efficacy in safely enhancing cardiopulmonary function while concurrently reducing negative emotional states and increasing mindfulness capacity. The intervention's clinical applicability is reinforced by three key mechanisms: (1) cognitive-behavioral enhancement through improved treatment adherence and executive functioning; (2) risk mitigation via real-time physiological monitoring that prevents exercise-induced complications; and (3) continuum of care achieved by integrating hospital-based rehabilitation protocols with community support systems. Notably, the program maintains high patient acceptability (92.68% satisfaction rate), which is crucial for sustaining cardiac rehabilitation outcomes.

### Limitations

This study had several limitations. The sample size was small, and the research period was short. The participants were all inpatients from a single center. Differences in healthcare levels and demographic characteristics and NYHA class, age groups, or gender may have affected the results. We recommend incorporating regression analyses that account for relevant covariates influencing SAS and SDS scores, FFMQ, 6MWD, and SFT outcomes. This would help establish the independent effect of the intervention while controlling for potential confounders. Future studies should expand the sample size to obtain more comprehensive data.

## Conclusion

In patients with atrial fibrillation and chronic heart failure, the Timing It Right-based mindful walking intervention group demonstrated significant improvements in mindfulness capacity, cardiopulmonary performance metrics, and negative emotion scores compared to standard care controls.

## Data Availability

The original contributions presented in the study are included in the article/Supplementary Material, further inquiries can be directed to the corresponding authors.
