# Supplementary figures and images for: The effectiveness of mindful walking based on the timing it right framework in patients with atrial fibrillation and chronic heart failure
Source: Front Cardiovasc Med. 2025 Jun 19;12:1587547. doi: 10.3389/fcvm.2025.1587547 (PMC12224207; doi:10.3389/fcvm.2025.1587547)

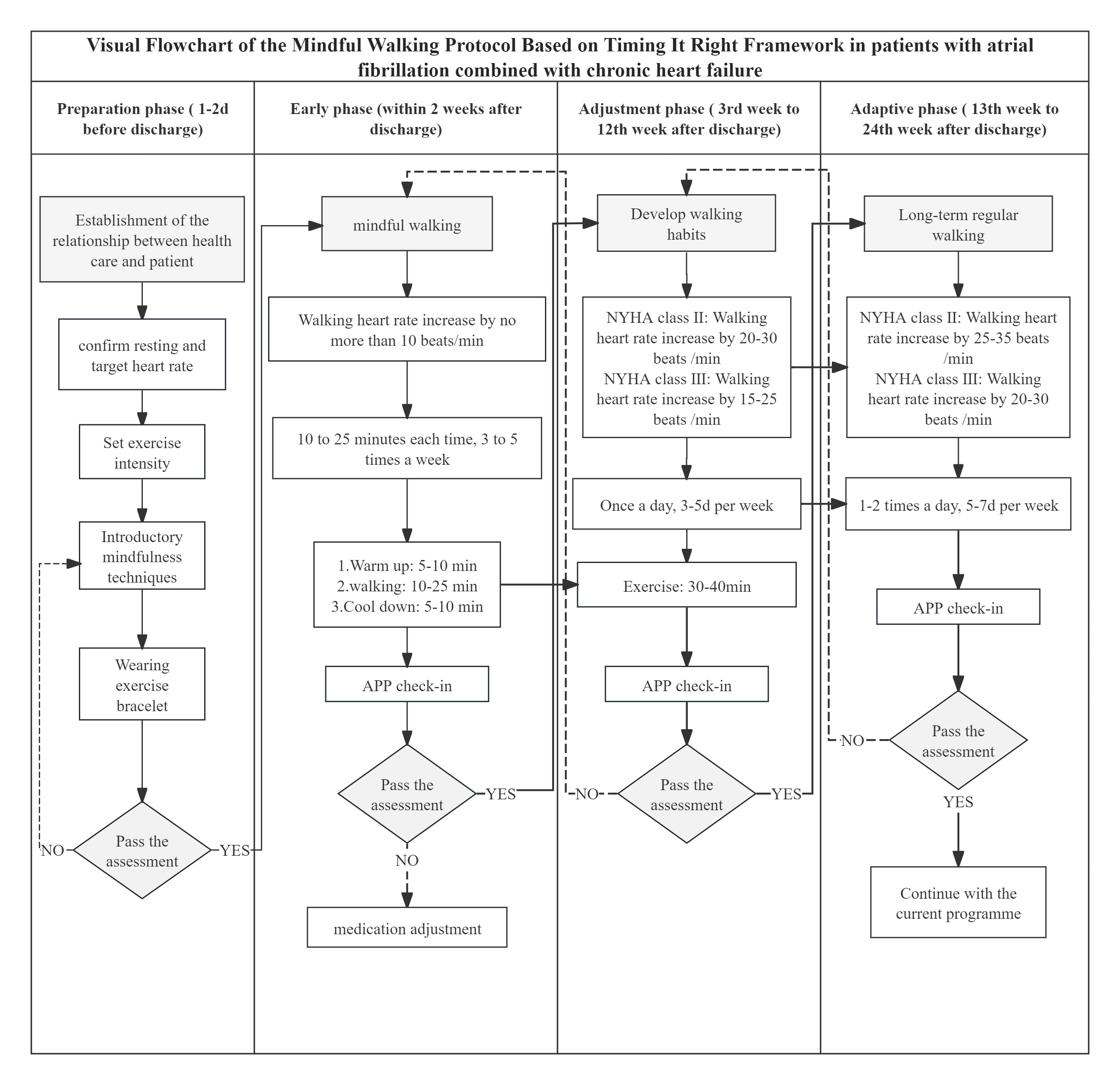

Supplement: Supplementary file 1 [file Image1.jpeg]
