# Supplementary material for: The effectiveness of mindful walking based on the timing it right framework in patients with atrial fibrillation and chronic heart failure
Source: Front Cardiovasc Med. 2025 Jun 19;12:1587547. doi: 10.3389/fcvm.2025.1587547 (PMC12224207; doi:10.3389/fcvm.2025.1587547)
Supplement: Supplementary file 2 [file Table1.docx]

| **Table S1 Subgroup analysis of the intervention effect of mindful Walking** | | | | | |
| --- | --- | --- | --- | --- | --- |
| Subgroup variable | Number | SAS (points,**Δ**) | SDS (points,**Δ**) | 6MWD (m,**Δ**) | FFMQ (points,**Δ**) |
| NYHA |  |  |  |  |  |
| NYHA Ⅱ | 35 | 15.54±7.11 | 12.42±8.41 | 62.00±74.43 | 5.69±12.27 |
| NYHA Ⅲ | 45 | 14.91±8.31 | 12.00±7.99 | 44.49±67.42 | 1.78±17.14 |
| P |  | 0.721 | 0.817 | 0.258 | 0.274 |
| Age |  |  |  |  |  |
| <60 | 45 | 15.96±7.43 | 12.67±7.77 | 59.8±68.50 | 3.56±16.16 |
| ≥60 | 35 | 14.32±8.27 | 11.53±8.76 | 3.67±14.31 | 44.38±74.73 |
| P |  | 0.319 | 0.553 | 0.421 | 0.964 |
| Gender |  |  |  |  |  |
| Male | 42 | 14.45±8.05 | 11.24±7.54 | 43.17±65.77 | 3.31±13.90 |
| Female | 38 | 16.00±7.47 | 13.24±8.70 | 62.08±75.32 | 3.68±16.78 |
| P |  | 0.377 | 0.275 | 0.234 | 0.913 |
